# Endogenous melatonin in critically ill patients

**DOI:** 10.1186/cc12388

**Published:** 2013-03-19

**Authors:** VS Salice, IG Galluccio, BS Salihovic, IP Piva, FM Marazzo, CV Villa, MT Taverna, MU Umbrello, GM Mistraletti, GI Iapichino

**Affiliations:** 1 Università degli Studi Milano, Milan, Italy

## Introduction

Melatonin could have a meaningful role in critically ill patients, because of its immunomodulatory, antioxidant and sleep regulation properties; it is reduced in critical illness. The purpose of this study is to describe the endogenous blood melatonin values in ICU patients and their correlation with clinical parameters.

## Methods

Seventy-three high-risk critically ill patients mechanically ventilated for >48 hours were enrolled. Blood samples for melatonin assay were collected between the 3rd and the 8th days of the ICU stay. Melatonin was determined by radioimmunoassay and ELISA. The peak and the area under the curve (AUC) calculated for each patient were correlated with the clinical parameters using the regression for quantiles test.

## Results

**Figure 1:**
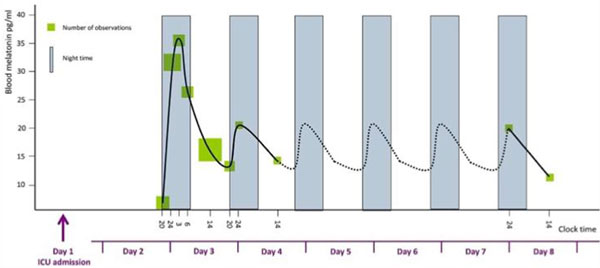


Endogenous melatonin was found lower in critically ill patients compared with healthy subjects (Figure 1), although it showed a great individual variability and it generally maintained a night-time increase. In the univariate analysis the peak was found related to: blood creatinine (*P *= 0.034); patients in coma (*P *= 0.024); hospital mortality (*P *= 0.016). The AUC was found related to: SAPS II (*P *= 0.047); creatinine (*P *0.001); AST (*P *0.001); ALT (*P *0.001); hospital mortality (*P *0.022). Peak and AUC were found higher in nonsurvivor patients.

## Conclusion

In accord with previous studies, the endogenous blood melatonin was found reduced in ICU patients. The higher melatonin peak in renal failure may be due to an increased distribution volume; greater AUC in patients with liver failure could be due to a less efficient removal of the hormone from the systemic circulation. The finding of increased peak and AUC in nonsurvivor patients could be due to a hormonal response increased by the body stress reaction, potentially similar to cortisol [1], or to a higher production of a physiological antioxidant [2] with a decreased ability to use it.
